# Professional Quality of Life of Public Health Nurses: Associations with Personal Characteristics, Job-Related Workload and Professional Support

**DOI:** 10.3390/ijerph23070922

**Published:** 2026-07-17

**Authors:** Ilona Karácsony, Gabriella Hideg-Fehér

**Affiliations:** 1 Department of Obstetrics and Prevention, Faculty of Health Sciences, Institute of Basic Health Sciences, Midwifery and Health Visiting, University of Pecs, Campus Szombathely, 7624 Pecs, Hungary; ilona.karacsony@etk.pte.hu; 2 Department of Sport Science, Faculty of Health Sciences, Institute of Physiotherapy and Sport Sciences, University of Pecs, 7624 Pecs, Hungary

**Keywords:** public health nurses, professional quality of life, ProQOL, burnout, secondary traumatization, professional support, supervision, case discussion

## Abstract

**Highlights:**

**Public health relevance—How does this work relate to a public health issue?**

- Public health nurses are exposed to high emotional and psychosocial workload, making their professional quality of life a critical public health concern.
- The study examines key determinants of professional well-being in a community-based preventive care setting.

**Public health significance—Why is this work of significance to public health?**

- Identifies personal, occupational, and support-related factors influencing burnout, secondary traumatization, and work satisfaction among public health nurses.
- Provides evidence that professional support systems play a central role in maintaining mental health and workforce sustainability.

**Public health implications—What are the key implications or messages for practitioners, policy makers and/or researchers in public health?**

- Expanding access to structured professional support (case discussions, supervision) is essential to reduce psychological burden and improve well-being.
- Strengthening training and organizational support systems may enhance resilience, prevent burnout, and improve the quality of primary healthcare services.

**Abstract:**

Background: The professional quality of life of public health nurses is a multidimensional construct associated with personal characteristics, job-related workload, and the availability of professional support. This study aimed to examine the associations between selected sociodemographic, occupational, and professional support factors and the professional quality of life of public health nurses in Hungary. Methods: A cross-sectional questionnaire-based study was conducted among public health nurses in Hungary using non-probability purposive sampling. The final sample consisted of 286 public health nurses who had been practicing for at least two years. In addition to self-developed items assessing sociodemographic characteristics, job-related factors, and professional support, the Professional Quality of Life Scale was applied. Data were analyzed using the Kolmogorov–Smirnov test, Mann–Whitney U test, Kruskal–Wallis test, and Spearman correlation analysis, with the level of significance set at *p* < 0.05. Results: Older age was associated with lower emotional overload resulting from work (r = −0.204, *p* = 0.001), lower secondary traumatization (r = −0.140, *p* = 0.018), and higher work satisfaction (r = 0.129, *p* = 0.029). Higher educational attainment was associated with lower emotional overload and burnout and higher work satisfaction. Public health nurses working in mixed districts reported higher emotional burden but also greater compassion satisfaction. Participation in professional training and case discussions was associated with more favourable positive indicators of professional quality of life, while supervision was associated with lower levels of burnout and secondary traumatization. Conclusions: The findings suggest that structured professional support systems, particularly regular case discussions, supervision, and continuing professional training, may contribute to maintaining professional well-being and workforce sustainability among public health nurses.

## 1. Introduction

Workplace stress, burnout, and compassion-related burden are major psychosocial challenges in helping professions, particularly in healthcare settings. Burnout is defined in the ICD–11 as an occupational phenomenon resulting from chronic workplace stress that has not been successfully managed [1]. Workplace stressors, such as role conflicts, excessive workload, administrative pressure, and insufficient resources, have been associated with poorer mental health and work-related psychological strain [2,3,4]. According to the Job Demands–Resources model, the combination of high job demands and insufficient resources increases the risk of burnout and work-related psychological burden [5].

The multidimensional model of burnout was originally developed by Maslach and Jackson [6], who described its three main components: emotional exhaustion, depersonalization, and reduced personal accomplishment. In helping professions, this framework has been extended by concepts such as compassion fatigue and secondary traumatization, which describe the negative psychological consequences of repeated exposure to the suffering, trauma, or crisis situations of others [7,8]. At the same time, helping work may also provide positive professional experiences, including work satisfaction and compassion satisfaction. Therefore, professional quality of life should be understood as a multidimensional construct that includes both positive and negative psychological consequences of helping work.

Public health nurses represent a particularly important professional group within community-based preventive healthcare. In Hungary, the public health nursing service has a long professional tradition and a specific role in maternal and child health care, school health activities, family support, prevention, and the child protection signalling system. Recent domestic studies indicate that the scope of tasks of public health nurses has expanded in recent years, particularly in relation to the child protection signalling system, which involves a high level of responsibility and emotional burden [9]. Professionals must not only provide maternal, child, and school health care, but also identify crisis situations, signal endangerment, cooperate with other professionals, and often manage conflict situations.

Domestic studies among healthcare professionals have shown that lack of professional recognition, excessive emotional involvement, and a strong sense of responsibility may contribute to psychological burden [10,11,12,13]. Community- and prevention-oriented professions, such as public health nursing, may therefore involve emotional and psychosocial demands that are comparable to those observed in other healthcare settings. Among public health nurses, increased workload, structural reorganization, and complex professional responsibilities have also been associated with chronic stress, burnout, and turnover intentions [14].

The professional quality of life of public health nurses may be closely related to the availability and quality of professional support. Several studies have emphasized the importance of collaboration between public health nurses and physicians, including effective communication, shared responsibility, and structured information exchange [15,16,17]. However, in domestic practice, regular forms of professional support—such as case discussions, supervision, professional training, and a stable substitution system—are often limited or unevenly available, despite their potential relevance for reducing psychological burden and supporting professional well-being.

Physical and psychological burden, continuous emotional work with clients and families, and overload resulting from systemic deficiencies may contribute to the deterioration of mental health among helping professionals [18]. One specific manifestation of this burden is secondary traumatization, which is also a central concept in the ProQOL framework [8]. The ProQOL framework provides a suitable basis for examining both the positive and negative psychological consequences of helping work. In the present study, we applied ProQOL-based dimensions to assess emotional overload resulting from work, work satisfaction, compassion satisfaction, secondary traumatization, and burnout in the context of public health nursing [19].

Although burnout, secondary traumatic stress, and professional quality of life have been widely examined among healthcare and helping professionals, considerably less is known about these phenomena in the specific context of public health nursing services operating in community-based preventive care systems. Public health nurses in Hungary fulfil a complex professional role that combines maternal and child health care, school health activities, family support, prevention, and participation in the child protection signalling system. Despite this complex workload, evidence remains limited regarding how personal characteristics, work-related factors, and access to professional support systems are associated with their professional quality of life. Therefore, the present study contributes to the literature by focusing on a nationally specific but internationally relevant professional group and by highlighting the role of structured professional support—particularly case discussions, supervision, and continuing professional training—in maintaining professional well-being.

## 2. Materials and Methods

### 2.1. Objectives

The aim of this study was to explore the professional quality of life of public health nurses and to examine its associations with selected sociodemographic characteristics, job-related factors, and forms of professional support. Professional quality of life was conceptualized as a multidimensional construct including both positive components, such as work satisfaction and compassion satisfaction, and negative components, such as emotional overload resulting from work, secondary traumatization, and burnout.

The study addressed the following research questions:

**RQ1:**  *How are selected sociodemographic characteristics, including age, marital status, place of residence, number of children, and educational level, associated with the dimensions of professional quality of life among public health nurses?*

**RQ2:**  *How are job-related factors, including work setting, years of professional experience, and long-term substitution work, associated with the positive and negative dimensions of professional quality of life?*

**RQ3:**  *How are different forms of professional support, including continuing professional training, case discussions, and supervision, associated with professional quality of life?*

Based on previous research on job demands, professional support, and burnout in helping professions, we formulated the following hypotheses:

**H1:**  *Higher age, stable partnership, and higher educational attainment are expected to be associated with more favourable indicators of professional quality of life, including higher work satisfaction and lower levels of emotional overload, secondary traumatization, and burnout.*

**H2:**  *Higher workload, particularly mixed district work and long-term substitution, is expected to be associated with higher emotional overload and secondary traumatization.*

**H3:**  *Greater access to professional support, including participation in professional training, case discussions, and supervision, is expected to be associated with higher work satisfaction and compassion satisfaction, as well as lower emotional overload, secondary traumatization, and burnout.*

### 2.2. Study Design and Participants

A quantitative, cross-sectional questionnaire-based study was conducted among public health nurses in Hungary. Participants were recruited using non-probability purposive sampling through online professional platforms commonly used by public health nurses. The inclusion criteria were higher education qualification in public health nursing and at least two years of professional practice. Public health nurses working part-time and those currently receiving treatment for mental health problems were excluded from the study.

A total of 302 responses were received. After applying the inclusion and exclusion criteria and screening for evaluable responses, the final analytical sample consisted of 286 public health nurses.

### 2.3. Measures

The questionnaire consisted of two main parts. The first part included self-developed items assessing sociodemographic characteristics, job-related factors, and forms of professional support. Sociodemographic variables included age, place of residence, marital status, number of children, and highest educational level. Job-related variables included work setting, years of professional experience, and participation in long-term substitution work. Work setting was categorized as district public health nursing, school public health nursing, or mixed district work. Mixed district work refers to a combined work setting in which the same public health nurse performs both district-based maternal, child, and family health visiting tasks and school health nursing duties. Professional support was assessed by questions on participation in continuing professional training, case discussions, and supervision.

The second part of the questionnaire assessed professional quality of life using the Hungarian version of the Professional Quality of Life Scale (ProQOL). Respondents indicated how often they had experienced each phenomenon during the previous 30 days on a five-point Likert scale, where 1 indicated “never” and 5 indicated “very often”. Higher scores indicated higher levels of the given dimension. (Table 1). The Hungarian version of the ProQOL used in the present study was validated by Kegye et al. [19]. In their validation study, exploratory factor analysis (EFA) identified an alternative structure consisting of five subscales, which differs from the original three-factor model. In order to better capture measurement validity and sample-specific characteristics in the Hungarian context, we applied this five-subscale approach in the present study. The internal consistency of the ProQOL-based subscales was acceptable to good for most dimensions: emotional overload resulting from work (Cronbach’s α = 0.888), work satisfaction (Cronbach’s α = 0.828), compassion satisfaction (Cronbach’s α = 0.812), and burnout (Cronbach’s α = 0.771). The reliability of secondary traumatization was lower but still acceptable for exploratory analysis (Cronbach’s α = 0.624).

### 2.4. Statistical Analysis

Statistical analyses were performed using IBM SPSS Statistics 28.0 (IBM Corp., Armonk, NY, USA). Descriptive statistics were used to characterize the sample and the ProQOL-based dimensions. The normality of the variables was assessed using the Kolmogorov–Smirnov test. As the distributions of the examined professional quality of life dimensions significantly deviated from normality, non-parametric statistical tests were applied.

Associations between continuous variables, such as age, years of professional experience, duration of substitution work, frequency of participation in professional training, and the dimensions of professional quality of life were examined using Spearman’s rank correlation analysis. Differences between two independent groups were tested using the Mann–Whitney U test, while differences across more than two groups were examined using the Kruskal–Wallis test. Statistical significance was set at *p* < 0.05.

The analyses were aligned with the research questions and hypotheses. Sociodemographic characteristics were examined in relation to H1, job-related factors in relation to H2, and forms of professional support in relation to H3.

Correlations below 0.30 were interpreted as weak associations. They were reported when statistically significant and when they were directly related to the predefined research questions and hypotheses; however, their practical relevance was interpreted cautiously.

### 2.5. Ethical Considerations

The study was conducted in accordance with the principles of the Declaration of Helsinki. Participation was voluntary and anonymous, and respondents provided informed consent before completing the questionnaire. No personally identifiable data were collected, and the responses were processed in aggregated form. Ethical approval was granted by the Regional Scientific and Research Ethics Committee of MEOK, Markusovszky University Teaching Hospital (protocol code: RTKB25/2024).

**Table 1:** Characteristics of the ProQOL-based subscales applied in the present study.

Subscale	Number of Items	What It Measures	Meaning of a High Score	Meaning of a Low Score
Emotional overload resulting from work	8	Psychological strain and emotional burden resulting from helping work	High emotional overload, chronic strain, excessive workload, exhaustion	Balanced workload, adequate resources, effective stress management
Work satisfaction	10	General and professional satisfaction, experiencing the meaningfulness of work	High commitment, positive work experiences, flexible coping, good resilience	Low satisfaction, frustration, reduced motivation, negative work experiences
Compassion satisfaction	5	Positive effects of the helper–client relationship, joy derived from helping, sense of well-being	Strong professional identity, sense of achievement, personal growth, positive feedback	Helping is not associated with positive experiences, lower commitment
Secondary traumatization	3	Taking on others’ trauma and problems, burden resulting from emotional involvement	Increased vulnerability, stress	Good boundary setting, lower emotional involvement in traumatic situations
Burnout	4	Negative consequences of caregiving, physical and psychological exhaustion	Emotional exhaustion, low mood, anxiety, low level of support	Stable psychological state, adequate energy level, effective coping

## 3. Results

### 3.1. Sample Characteristics and Professional Quality of Life

The final sample consisted of 286 public health nurses. The mean age of the respondents was 39.93 years (SD = 10.73; range: 25–65). The sample included participants with different residential backgrounds, marital statuses, educational levels, and work settings. The main sociodemographic and job-related characteristics of the sample are presented in Table 2.

Overall, the sample was heterogeneous in terms of place of residence and work setting, while the majority of participants were married or living in a cohabiting relationship and held a college degree. Nearly one-third of respondents reported long-term substitution work, indicating that additional workload was present in a considerable proportion of the sample.

Professional quality of life was assessed using the ProQOL-based dimensions applied in the present study. These dimensions included positive components, such as work satisfaction and compassion satisfaction, and negative components, such as emotional overload resulting from work, secondary traumatization, and burnout. Table 3 presents the descriptive statistical values of these dimensions, including mean, standard deviation, minimum, and maximum values.

Based on the normality test performed using the Kolmogorov–Smirnov test, *p* < 0.05 was confirmed for all subfactors; therefore, non-parametric tests were applied in further analyses.

Regarding age, older respondents reported significantly lower emotional overload resulting from work (r = −0.204, *p* = 0.001) and lower secondary traumatization (r = −0.140, *p* = 0.018), while work satisfaction increased significantly with age (r = 0.129, *p* = 0.029). No significant associations were found between age and compassion satisfaction (r = 0.049, *p* = 0.406) or burnout (r = −0.084, *p* = 0.157).

Educational level was also associated with several dimensions of professional quality of life. Compared with public health nurses holding a college degree, those with a university degree reported lower emotional overload resulting from work (MD = 1.15; U = 7081.5, Z = −2.204, *p* = 0.028) and lower burnout (MD = 2.79; U = 6686.0, Z = 2.83, *p* = 0.0045), while their work satisfaction was significantly higher (MD = 2.63; U = 6939.0, Z = −2.43, *p* = 0.015). No significant differences were observed between educational groups in compassion satisfaction (U = 8176.0, Z = −0.486, *p* = 0.627) or secondary traumatization (U = 7279.0, Z = −1.913, *p* = 0.056).

Marital status was significantly associated with all examined dimensions of professional quality of life. Respondents living in a marital or cohabiting relationship reported higher levels of work satisfaction and compassion satisfaction, while negative dimensions, including emotional overload resulting from work, secondary traumatization, and burnout, were lower compared with respondents who were single, divorced, or widowed. The group differences were statistically significant for work satisfaction (H = 31.62, *p* < 0.001), compassion satisfaction (H = 22.69, *p* < 0.001), emotional overload resulting from work (H = 22.30, *p* < 0.001), secondary traumatization (H = 17.36, *p* = 0.001), and burnout (H = 35.73, *p* < 0.001).

### 3.2. Work-Related Factors and Professional Quality of Life

Emotional overload resulting from work differed significantly by work setting (H(2) = 13.908, *p* = 0.001). The highest mean value was observed among public health nurses working in mixed districts (M = 27.86), followed by district public health nurses (M = 24.31) and school public health nurses (M = 21.57).

Compassion satisfaction also differed significantly by work setting (H(2) = 6.81, *p* = 0.033). Mean values were higher among public health nurses working in mixed districts (M = 20.00) and in school public health nursing (M = 19.43) compared with district public health nurses (M = 18.98). No statistically significant differences were found by work setting in work satisfaction (H(2) = 1.13, *p* = 0.56), secondary traumatization (H(2) = 4.16, *p* = 0.125), or burnout (H(2) = 3.592, *p* = 0.166).

The respondents had practiced their profession for an average of 15.98 years (SD = 11.09; range: 2–44 years). Years of professional experience were not significantly associated with any of the examined dimensions of professional quality of life: emotional overload resulting from work (r = −0.078, *p* = 0.190), work satisfaction (r = 0.053, *p* = 0.371), compassion satisfaction (r = −0.050, *p* = 0.400), secondary traumatization (r = 0.015, *p* = 0.806), or burnout (r = 0.058, *p* = 0.327).

A total of 29.3% of public health nurses reported performing long-term substitution work, with an average duration of 3 years. Compared with those who did not perform substitution work, no statistically significant differences were found between the two groups in emotional overload resulting from work (U = 7695.5, Z = −1.239, *p* = 0.215), secondary traumatization (U = 7808.0, Z = −1.074, *p* = 0.283), or burnout (U = 8473.5, Z = −0.017, *p* = 0.987). However, compassion satisfaction was significantly higher among those performing substitution work (MD = 1.66; U = 6070.5, Z = −3.810, *p* < 0.001), while work satisfaction did not differ significantly between the two groups (substitution: M = 35.08; no substitution: M = 33.14; U = 7573.0, Z = −1.433, *p* = 0.152).

Among public health nurses performing substitution work, the duration of substitution was significantly and moderately negatively associated with secondary traumatization (r = −0.395, *p* < 0.001). This result suggests that longer duration of substitution was associated with lower levels of secondary traumatization in this subgroup. No significant associations were found between the duration of substitution and emotional overload resulting from work (r = −0.204, *p* = 0.062), work satisfaction (r = 0.097, *p* = 0.380), or burnout (r = −0.037, *p* = 0.741).

### 3.3. Professional Support and Professional Quality of Life

Professional support was examined through three indicators: participation in continuing professional training, case discussions, and supervision. Public health nurses participated in professional training and lectures on average twice per year (M = 2.05, SD = 1.17). The frequency of participation in professional training was significantly negatively associated with emotional overload resulting from work (r = −0.150, *p* = 0.011), and significantly positively associated with compassion satisfaction (r = 0.182, *p* = 0.002) and work satisfaction (r = 0.296, *p* < 0.001). No significant associations were found between participation in professional training and secondary traumatization (r = −0.020, *p* = 0.736) or burnout (r = 0.055, *p* = 0.350).

Participation in case discussions was assessed using four response categories: “yes, regularly”, “yes, occasionally”, “no, because it is not available”, and “no”. A total of 41 respondents participated regularly (14.3%), 46 participated occasionally (16.1%), 181 reported that case discussions were not available to them (63.3%), and 18 reported that they did not participate in such activities (6.3%). Differences in the ProQOL-based dimensions by participation in case discussions are presented in Table 4.

Significant differences by participation in case discussions were observed for emotional overload resulting from work (H(3) = 16.562, *p* = 0.001), work satisfaction (H(3) = 59.131, *p* < 0.001), and compassion satisfaction (H(3) = 44.063, *p* < 0.001). Respondents who participated in case discussions regularly or occasionally reported lower emotional overload than those for whom case discussions were not available or who did not participate. Work satisfaction was highest among those participating regularly. Compassion satisfaction was also highest among those participating regularly and lowest among those reporting no access to case discussions. No significant differences were found for secondary traumatization (H(3) = 3.839, *p* = 0.279) or burnout (H(3) = 5.130, *p* = 0.162).

**Figure 1:**
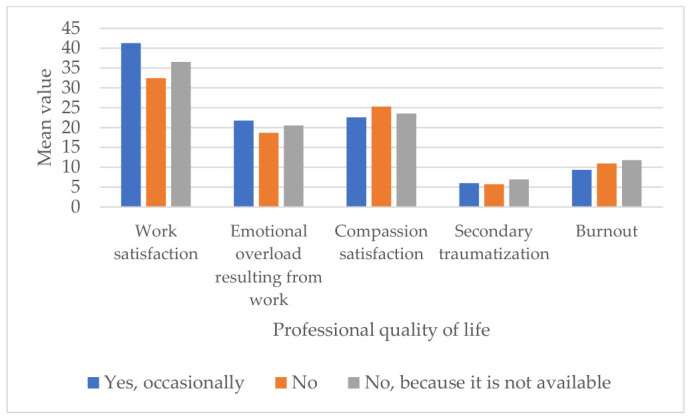
Mean values of professional quality of life by participation in supervision (N = 286).

Regarding supervision, none of the respondents reported regular participation. A total of 8.74% participated in supervision occasionally, while 91.26% did not participate at all, either because supervision was not available or because they did not use this opportunity. Participation in supervision was significantly associated with several dimensions of professional quality of life. Respondents who participated in supervision reported lower secondary traumatization (H(2) = 12.65, *p* = 0.002) and burnout (H(2) = 11.63, *p* = 0.003), as well as higher work satisfaction (H(2) = 42.62, *p* < 0.001) and compassion satisfaction (H(2) = 21.19, *p* < 0.001). No statistically significant difference was observed for emotional overload resulting from work (H(2) = 4.02, *p* = 0.134). The mean values by participation in supervision are presented in Figure 1.

## 4. Discussion

The aim of this study was to examine the professional quality of life of public health nurses and its associations with selected sociodemographic characteristics, job-related factors, and forms of professional support. Overall, the findings indicate that professional quality of life among public health nurses is shaped by a complex pattern of personal, occupational, and organizational factors. The results partly supported the first hypothesis, as older age and higher educational attainment were associated with more favourable professional quality of life indicators. The second hypothesis was also partly supported: mixed district work was associated with higher emotional overload, while substitution work showed a more complex pattern, being associated with higher compassion satisfaction but not with higher burnout or emotional overload. The third hypothesis received the strongest support, as participation in professional training, case discussions, and supervision was consistently associated with more favourable indicators of professional quality of life.

Regarding sociodemographic characteristics, age appeared to be associated with a more favourable pattern of professional quality of life. Older respondents reported lower emotional overload and secondary traumatization, as well as higher work satisfaction. This may suggest that professional and life experience, more established coping strategies, and clearer personal boundaries could help reduce emotional strain in public health nursing. Similar findings have been reported in healthcare workers, where older age and more favourable occupational conditions were associated with lower burnout risk [20,21,22,23]. However, age was not significantly associated with compassion satisfaction or burnout, indicating that these dimensions may depend not only on individual maturity or experience, but also on organizational and contextual factors.

Educational level also showed a favourable association with professional quality of life. Public health nurses with a university degree reported lower emotional overload and burnout and higher work satisfaction. This finding may reflect the role of higher education in strengthening professional competence, autonomy, self-efficacy, and confidence in managing complex professional situations. Similar conclusions were drawn by La Torre et al. [20], who identified higher education, older age, and better physical and mental health as factors associated with lower burnout risk. At the same time, as the cross-sectional design does not allow causal interpretation, these associations should be interpreted cautiously.

Marital status was also associated with professional quality of life. The most favourable pattern was observed among married respondents, who reported higher positive indicators and lower negative indicators. This may point to the potential role of stable social support in buffering work-related psychological burden. This interpretation is consistent with the Job Demands–Resources model, which emphasizes the relevance of available resources in reducing the negative consequences of high job demands [5]. Nevertheless, the differences between marital status groups should be interpreted with caution, as partnership status alone does not fully capture the quality, availability, or perceived adequacy of social support.

Work setting was also related to professional quality of life. Public health nurses working in mixed districts reported the highest level of emotional overload. This finding may be explained by the broader range of tasks, the diversity of client groups, and the greater role complexity associated with providing both district and school public health nursing care. This interpretation is consistent with the Job Demands–Resources model, which emphasizes that high job demands combined with insufficient resources may contribute to psychological strain and burnout [5,9]. At the same time, compassion satisfaction was also higher among those working in mixed districts and in school public health nursing. This suggests that more diverse or child- and family-centred work may not only increase emotional burden, but may also provide meaningful professional experiences. This dual pattern is consistent with the ProQOL framework, which conceptualizes helping work as involving both negative and positive professional experiences [8,19].

Substitution work showed a similarly complex pattern. Public health nurses performing long-term substitution did not report significantly higher emotional overload, secondary traumatization, or burnout than those not performing substitution work, but they showed higher compassion satisfaction. This may indicate that additional helping responsibilities can also be accompanied by positive professional meaning and perceived usefulness. This interpretation is consistent with the ProQOL framework, which emphasizes that helping work may involve both burden and satisfaction, depending on professional resources, emotional demands, and perceived meaning [8,22]. Interestingly, among those performing substitution work, longer duration of substitution was negatively associated with secondary traumatization. One possible explanation is that professionals who continue substitution work for a longer period may develop more effective coping strategies or may represent a more resilient subgroup. However, this interpretation remains tentative and should be examined in future studies.

Professional support emerged as one of the most important areas of the study. Participation in professional training was associated with lower emotional overload and higher work satisfaction and compassion satisfaction. This finding suggests that continuing professional development may provide not only updated professional knowledge, but also a sense of competence, professional security, and connection to the wider professional community. The importance of structured professional development and collaboration is also supported by previous studies on public health nursing and healthcare work, which emphasize the role of professional cooperation, information exchange, and organizational support [15,16]. However, participation in training was not significantly associated with burnout or secondary traumatization, which may indicate that these deeper psychological burdens require more targeted forms of support than training alone. This interpretation is consistent with previous findings showing that burnout among healthcare professionals is associated with workload, emotional strain, psychological variables, and insufficient resources [12,21,22].

Case discussions also appeared to be closely related to professional quality of life. Respondents who participated in case discussions regularly or occasionally reported lower emotional overload, while work satisfaction was highest among those participating regularly. This suggests that case discussions may function as an important form of reflective professional support, providing opportunities to share difficult cases, reduce professional isolation, and strengthen problem-solving in emotionally demanding situations. This is particularly relevant in public health nursing, where professionals frequently encounter complex family, social, and child protection problems requiring interprofessional cooperation and structured information exchange [14,15,16]. The fact that most respondents reported that case discussions were not available highlights an important organizational gap in the current support system.

Supervision showed a similarly favourable pattern. Although only a small proportion of respondents participated in supervision, those who did reported lower secondary traumatization and burnout, as well as higher work satisfaction and compassion satisfaction. This finding is particularly important because supervision is specifically designed to support emotional processing, professional reflection, and the management of difficult client-related experiences. Previous research on burnout and professional quality of life among healthcare professionals has emphasized the importance of organizational support, emotional support, and structured professional resources in reducing psychological burden [18,24,25]. The limited availability of supervision therefore represents a relevant organizational issue, especially in a profession characterized by high responsibility, emotional involvement, and exposure to family crises and child protection concerns.

**Practical implications**

The findings have several practical implications for public health nursing services. First, the high proportion of respondents reporting no access to case discussions or supervision indicates that structured professional support is not equally available for all public health nurses. Regular case discussions could provide a low-threshold organizational tool for reducing emotional overload, strengthening professional reflection, and supporting problem-solving in complex family and child protection cases. Second, supervision should be considered as a targeted form of emotional and professional support, particularly for professionals exposed to high responsibility, emotionally demanding situations, and secondary traumatization. Third, continuing professional training should not only focus on professional knowledge and regulatory updates, but should also include topics related to stress management, boundary setting, communication in conflict situations, and self-care. These interventions may contribute to workforce sustainability and the prevention of burnout among public health nurses.

**Limitations**

Several limitations should be acknowledged. First, the cross-sectional design does not allow causal conclusions; therefore, the results should be interpreted as associations rather than effects. Second, the study used non-probability purposive sampling, which limits the generalizability of the findings. Third, all data were based on self-reported questionnaire responses, which may be affected by response bias and subjective interpretation. Fourth, public health nurses currently receiving treatment for mental health problems were excluded from the study. This criterion was applied to reduce potential confounding related to ongoing mental health treatment; however, it may also have excluded a particularly vulnerable subgroup and may therefore have led to an underestimation of psychological burden. Finally, the analyses were primarily based on bivariate statistical methods and did not control for potential confounding variables in multivariable models. Future studies should address this limitation using larger samples and more complex analytical designs.

**Future research**

Future research should examine the professional quality of life of public health nurses using longitudinal and multivariable study designs in order to clarify the independent and combined role of personal, occupational, and organizational factors. Further studies should also investigate the quality, frequency, and perceived usefulness of case discussions and supervision, rather than only their availability. Qualitative research could provide deeper insight into how public health nurses experience emotional overload, secondary traumatization, and professional support in everyday practice. Finally, intervention studies are needed to evaluate whether structured supervision, regular case discussions, and targeted professional training can reduce psychological burden and improve professional well-being over time.

## 5. Conclusions

This study examined the professional quality of life of public health nurses in relation to selected sociodemographic characteristics, job-related factors, and forms of professional support. The findings suggest that professional quality of life in this professional group is associated with a complex pattern of personal, occupational, and organizational factors. Older age, higher educational attainment, and marital status were related to more favourable indicators of professional quality of life, while mixed district work was associated with higher emotional overload. Substitution work showed a more nuanced pattern, as it was associated with higher compassion satisfaction but not with higher burnout or emotional overload.

The most consistent findings concerned professional support. Participation in professional training, case discussions, and supervision was associated with more favourable professional quality of life indicators, particularly higher work satisfaction and compassion satisfaction and, in the case of supervision, lower burnout and secondary traumatization. These results highlight the importance of structured professional support systems in public health nursing. Expanding access to regular case discussions, supervision, and targeted continuing professional training may contribute to maintaining professional well-being, reducing psychological burden, and supporting workforce sustainability among public health nurses.

## Figures and Tables

**Table 2:** Sociodemographic and job-related characteristics of the sample (N = 286).

Variables	Mean	Range
**Age**	39.93 ± 10.73 years	25–65 years
**Number of children**	1.39 ± 1.11	0–4
**Years of professional experience**	15.98 ± 11.09 years	2–44 years
	**absolute frequency (*n*)**	**relative frequency (%)**
**Number of children**		
**No children**	86	30.1%
**One child**	55	19.2%
**Two children**	99	34.6%
**Three children**	40	14.0%
**Four children**	6	2.1%
**Gender**		
**Women**	286	100%
**Place of residence**		
**Capital city**	39	13.6%
**County seat**	55	19.2%
**Town**	60	21%
**Village**	132	46.2%
**Marital status**		
**Married**	164	57.34%
**Cohabiting partnership**	61	21.33%,
**Single**	35	12.24%
**Divorced**	23	8.04%,
**Widowed**	3	1.05%
**Educational attainment**		
**College degree**	202	70.6%
**University degree**	84	29.4%
**PhD degree**	0	0%
**Work setting**		
**District public health nurse**	193	67.4%
**Mixed district public health nurse**	72	24,4%
**School public health nurse**	21	7.3%
**Long-term substitution work**		
**Yes**	84	29.3%
**No**	202	70.7%

**Table 3:** Descriptive statistical values of the ProQOL-based dimensions applied in the present study (N = 286).

	Maximum Possible Score	Mean	SD	Min	Max
Emotional overload resulting from work	40	22.25	7.39	8	39
Work satisfaction	50	33.71	7.69	11	47
Secondary traumatization	25	19.17	3.82	8	25
Compassion satisfaction	15	5.87	2.13	3	11
Burnout	20	10.85	3.13	5	18

**Table 4:** ProQOL-based dimensions by participation in case discussions (N = 286).

ProQOL-Based Dimension	Yes, Regularly (*n* = 41) M ± SD	Yes, Occasionally (*n* = 46) M ± SD	No, Because It Is Not Available (*n* = 181) M ± SD	No (*n* = 18) M ± SD	Kruskal–Wallis Test
Emotional overload resulting from work	19.32 ± 7.48	19.59 ± 7.34	23.62 ± 7.23	22.00 ± 5.60	H(3) = 16.562, *p* = 0.001
Work satisfaction	41.02 ± 6.35	34.50 ± 8.51	31.68 ± 6.86	35.50 ± 5.72	H(3) = 59.131, *p* < 0.001
Compassion satisfaction	22.29 ± 2.91	19.37 ± 4.61	18.31 ± 3.53	20.17 ± 2.41	H(3) = 44.063, *p* < 0.001
Secondary traumatization	6.22 ± 2.03	5.67 ± 1.74	5.88 ± 2.26	5.50 ± 2.12	H(3) = 3.839, *p* = 0.279
Burnout	9.93 ± 2.49	10.48 ± 3.48	11.20 ± 3.19	10.33 ± 2.57	H(3) = 5.130, *p* = 0.162

## Data Availability

The data presented in this study are available on request from the corresponding author. The data are not publicly available due to privacy and ethical restrictions.

## Abbreviations

The following abbreviations are used in this manuscript:

ICD	International Classification of Diseases
JD–R model	Job Demands–Resources model
M	Mean
MAVE	Hungarian Association of Public Health Nurses
MD	Mean Difference
MESZK	Hungarian Chamber of Healthcare Professionals
MVSZSZ	Hungarian Association of Public Health Nurses (Professional Association)
ProQOL	Professional Quality of Life Scale
SD	standard deviation
WHO	Word Health Organization
